# mSphere of Influence: Comprehensive Genetic Analysis

**DOI:** 10.1128/mSphere.00181-20

**Published:** 2020-03-11

**Authors:** Jason M. Peters

**Affiliations:** a Pharmaceutical Sciences Division, School of Pharmacy, University of Wisconsin—Madison, Madison, Wisconsin, USA; b Department of Bacteriology, University of Wisconsin—Madison, Madison, Wisconsin, USA; c Department of Medical Microbiology and Immunology, University of Wisconsin—Madison, Madison, Wisconsin, USA; d Great Lakes Bioenergy Research Center, Wisconsin Energy Institute, University of Wisconsin—Madison, Madison, Wisconsin, USA

**Keywords:** *Bacillus subtilis*, CRISPR, *Saccharomyces cerevisiae*, gene networks, genetic interactions, genetics, systems biology

## Abstract

Jason M. Peters works in the fields of antibiotic resistance and biofuel production. In this mSphere of Influence article, he reflects on how the paper “A global genetic interaction network maps a wiring diagram of cellular function” by Costanzo et al. (Science 353:aaf1420, 2016, https://doi.org/10.1126/science.aaf1420) has impacted his work by highlighting the power of gene networks to uncover new biology.

## COMMENTARY

A fundamental question in genetics is “How do genes work together to carry out complex cellular processes?” Gene networks promise to answer this question by illuminating how interactions between genes contribute to pathways and, ultimately, higher-order processes. We can build gene networks by systematically probing phenotypes and using the patterns across these phenotypes to link genes that have shared function. One outstanding example of this approach is found in a longstanding effort by the Andrews, Boone, and Myers labs to comprehensively map the genetic interaction network of the yeast Saccharomyces cerevisiae. Over a series of impactful publications (1–3), these labs progressed toward their shared goal, culminating in the paper “A global genetic interaction network maps a wiring diagram of cellular function” (3). In a *tour de force*, the authors constructed a gene interaction network from ∼23 million gene-gene interaction pairs, uncovering new functions for poorly annotated genes and new additions to established pathways. Some of the results I found most intriguing—e.g., that essential genes are enriched for interactions and serve as genetic “hubs”—could be determined only at the network level.

A classic adage in genetics is “You get what you screen for.” Therefore, the authors performed a massive, open-ended screen to uncover as much new biology as possible. Their double mutant screen involved pairwise combinations of nearly all genes in S. cerevisiae. They took advantage of two key resources available to S. cerevisiae researchers: deletion libraries of all nonessential genes (4) and libraries of temperature-sensitive alleles of essential genes collected from the yeast community or generated in the authors’ labs (5). Due to the incredible scale of the screen, double mutant construction and analysis were largely automated; the authors carried out matings by robotically pinning cells on agar plates that selected for both mutant alleles and then calculated fitness scores for these double mutants based on colony size extracted from plate images. Extensive normalization made data obtained through these large-scale growth experiments interpretable—systematic experimental noise from variations within/between plates and across time can dominate the signal if not removed. Finally, the authors built their gene network by calculating the difference between the expected fitness of the double mutant based on phenotypes of the single mutants (6) and the actual phenotype of the double mutant and then correlating these differences across genes. Genes with similar interaction profiles above a statistical cutoff were connected in the network.

In many ways, the work of Costanzo et al. (3) is a prime example of a broader trend in genetics and in my own work away from thinking about individual genes and toward thinking about cellular systems and networks. This is immediately apparent from the striking network visualizations that comprise the first few figures of the paper; in showing clusters of genes color coded by process, these figures quickly convey the ability of a gene network to recapitulate decades of yeast biology in a single, albeit massive experiment. What the figures do not show are the interactions between individual genes (although that can be viewed online at thecellmap.org/). I have to admit that, as a bacterial geneticist, I am very jealous of these networks and I would like to have them for my own favorite bacterial species. In particular, I want to know the answers to a number of questions raised by this paper when applied to bacteria. Are “rules” about gene interactions—such as there being many more negative interactions than positive ones—maintained in a different domain of life? Do essential genes still act as network hubs, and how are these hubs conserved across species? What might we learn about gene function from applying a genetic interaction approach to poorly characterized species? Above all, this series of papers taught me that having a far-reaching goal and casting a wide net were not only possible but could actually inform new ways of thinking about biology at a different scale.

With their comprehensive gene network, the Andrews, Boone, and Myers labs provided a template for future work in network and systems biology. For instance, my own network study of essential genes in Bacillus subtilis (7) was strongly influenced by their approach. Although the automated mating strategy used by the authors is time/resource intensive and species-specific, new approaches—such as using CRISPR interference (CRISPRi) to define genetic interactions in pooled format (8, 9)—may increase throughput and decrease costs, democratizing the comprehensive study of gene networks in more species. The work of Costanzo et al. opened the door to genome-scale interrogation into how genes work together to carry out cellular processes. I am excited to see future efforts that address the questions raised by this work throughout the tree of life.
